# ‘At the heart of the community’ – a Somali woman’s experience of ‘alignment’ of support to escape social isolation in pregnancy and early motherhood

**DOI:** 10.1080/17482631.2024.2439467

**Published:** 2024-12-17

**Authors:** Tom Allport, Hannah Briggs, Fatumo Osman

**Affiliations:** a Centre for Academic Child Health, Bristol Medical School, University of Bristol, Bristol, UK; b Community Children’s Health Partnership, Sirona Care & Health, Bristol, UK; c School of Health and Welfare, Dalarna University, Bristol, Sweden

**Keywords:** Pregnancy, motherhood, migration, Somali, alignment, resilience, community, social support

## Abstract

**Purpose:**

Stresses in pregnancy and early motherhood can affect women’s health and wellbeing, and babies’ development. Migrant women face compounding stressors from the intersection of gender, race, social class, migration, and language. We explored one Somali woman’s experience of pregnancy and the transition to motherhood, following migration to an urban environment in the Global North, aiming to understand resilience in this specific socio-cultural context.

**Methods:**

This case study used interpretative phenomenological analysis of a single two-hour semi-structured interview with a Somali woman in the UK to explore how this experience may have relevance for communities and practitioners in the Global North.

**Results:**

We identified two overarching themes in this woman’s experience: “vicious” and “virtuous” circles, attempting to make sense of her experience of isolation and lack of wellbeing, and subsequent confidence, engagement, and community-building.

**Conclusions:**

An experience of “alignment” in social relationships appeared to make possible the shift from “vicious” to “virtuous” circle, which enabled escape from social isolation. This account of transformation—from social isolation to community contribution—underlines the role of community organizations facilitating positive social networks and peer support during pregnancy and early motherhood.

## Introduction

Pregnancy and early motherhood can bring stress and affect women’s health and wellbeing, and babies’ development (CAO-LEI et al., 2020; Madigan et al., 2018; Rogers et al., 2020; Staneva et al., 2015; Viswasam et al., 2019; Woody et al., 2017).

Migrant women face compounding stressors arising from the intersection of gender, race, social class, immigration, and language. They are at high risk of adverse pregnancy and perinatal health outcomes, with care obstructed by structural, organizational, social, personal, and cultural barriers (Benza & Liamputtong, 2014; Giscombe et al., 2020; Heslehurst et al., 2018; Pangas et al., 2019), and challenges with communication and access to healthcare (Degrie et al., 2017; Fair et al., 2020; Heslehurst et al., 2018).

Many Somalis have fled conflict (UNHCR, 2024) and represent one of the UK’s largest disadvantaged migrant groups (Office for National Statistics, 2023; Wallis, 2018), bringing strong community and family values (Evason, 2019; Lightfoot et al., 2016), which can present challenges adjusting to more individualistic Western contexts; 5% of Bristol’s children are of Somali origin. Somali women in the Global North experience a similar range of challenges during pregnancy and childbirth to other disadvantaged and marginalized migrants—prominently lack of information, conflicting recommendations, language and cultural barriers, and challenges to confident, trusting relationships with professionals which all may contribute to difficulties navigating healthcare systems, and adverse experiences (Bains et al., 2021; Clark et al., 2018; Jacoby et al., 2015; Wallmo et al., 2020). As with other migrants, little attention has been paid to more holistic, personal and social needs.

There are multiple barriers to service engagement with Somali and other Northern and Eastern African migrants in healthcare systems in the Global North (Oommen et al., 2024; Sharma et al., 2023). In light of this, attempts have been made to tailor the services offered. Key elements of more acceptable and accessible models can be summarized as: culturally responsive care, continuity of care, effective communication, psychosocial and practical support, support to navigate systems, and flexible and accessible services (Madeira et al., 2019; Rogers et al., 2020). Reports have generally focused on the delivery of health services, often lacking attention to broader (non-healthcare-related) experiences, as well as the potential role of community rather than professional support. However, attention has been paid to the challenge of constructing maternal identity across cultures, and the importance of social support (Pangas et al., 2019; Winter et al., 2023).

Using an interpretative phenomenological approach to the experience of one Somali migrant woman, we aimed to explore what helped, and what challenged her experience of pregnancy and the transition to motherhood, looking to understand resilience in a specific socio-cultural context (Bronfenbrenner, 1986; Peyton & Wisniewski, 2019; Ungar & Theron, 2020), and to consider how both practitioners and communities in the Global North can best support Somali migrant women during this time.

## Materials and methods

### Study design and rationale

We report a single case study by semi-structured interview with interpretative phenomenological analysis.

Interpretative Phenomenological Analysis (IPA) is well suited to individual interviews of participants, with its idiographic attention to the individual and the particular (Eatough & Smith, 2006; Glasscoe & Smith, 2008; Smith, 2004). It explores participant’s experiences and meanings while accounting for researchers’ perceptions and interpretations (Noon, 2018; Smith et al., 1999). Single case studies can contribute “a nuanced view of reality” (Flyvbjerg, 2006), understanding of mechanisms (Pietkiewicz & Smith, 2014; Smith, 1996, 2004), and theoretical generalization, in the sense that case study, using robust research process and rich, clear interpretation (Lewis & Ritchie, 2003) can develop new theory, to “give weight to the concepts required to frame what is special about these states for the people involved” (Radley & Chamberlain, 2012).

Seeking to understand resilience processes, we took an appreciative, asset-based perspective (BONMATÍ-Tomás et al., 2016; Cooperrider & Whitney, 2005; Masten, 2001; O'Dougherty Wright et al., 2013).

### Participant characteristics and recruitment

A participant was sought purposively through an invitation presented to a local Somali women’s group by a (Somali) associate in our research and advocacy partnership, seeking a Somali woman who had been previously pregnant in the UK within the last 3 years.^1^ The participant identified from this was in her thirties, had two children, came to the UK about 11 years previously, completed secondary (high) school studies in the UK, and was not in paid employment.

### Semi-structured interview, data collection, participant and peer feedback

Semi-structured interviews build rapport, help conversation flow, and permit flexibility while ensuring key topics are covered (Mack et al., 2005; Smith, 1995). A semi-structured interview schedule was developed to address topics identified from literature review and stakeholder meetings, including aspects of daily routine, support network, wellbeing & stress, and the experience of the birth and transition to motherhood (see Appendix).

Due to Covid-19, the in-depth (2 hour) interview was undertaken by telephone (in April 2020), with the participant at home. The interview was audio-recorded; no translator was required. The interview was conducted by a medical student studying an “intercalated” BSc degree in Global Health, with live supervision during the interview by an experienced Paediatrician and Researcher to ensure safe and ethical practice.

Ethical approval was gained from the University of Bristol Faculty of Health Sciences Research Ethics Committee—ref 101,743.

The participant read and fed back positively on the findings and discussion in this article, not suggesting any additions or alterations.

### Data analysis and reflexivity

Interpretative Phenomenological Analysis was undertaken according to published guidance (Pietkiewicz & Smith, 2014; Smith et al., 1999a, 2021). One of the authors transcribed the interview, listening and reading multiple times to gain a close familiarity with the text, before starting the analysis. During the first cycle of annotation through the transcript, important points and thoughts were noted in one margin; in the second cycle an inductive approach was taken to generate experiential statements attempting to characterize how each short section of text was addressing the research question, which were noted in the other margin. Experiential statements were clustered together into emerging themes as connections between them were established, and overarching theme titles were given to clusters of these themes. This was continued until a hierarchy of themes was able to represent the analytical process of the whole interview, with broad, overarching themes drawing together more specific, sub-ordinate themes. Throughout the process, the transcript was iteratively referred to, to ensure experiential statements and themes at each level of abstraction reflected the participant’s meaning and experience, weaving the analysis between text/participant and interpretation/researcher.

Analytic process and results were discussed with and audited by an experienced IPA researcher, following usual IPA practice (Smith et al., 2021), and with a practitioner-researcher of Somali origin, who together have over 50 years’ experience of working and researching with Somali families in the Global North. We have since presented these findings to a number of groups of Somali community members, who have responded to the themes presented as very much resonating with aspects of their own and their peers’ experiences. This study forms part of a 10-year body of collaborative, co-produced approach to research and social/environmental change for migrant families in the UK that we have called “Find your village”.

Researchers attempted to maintain self-reflexivity throughout (Braun & Clarke, 2006; Finlay, 2002; Kelly et al., 2010). The interview and primary analysis were undertaken by a medical student; the co-authors have over 50 years’ combined experience of practice and research in multi-cultural health and community settings.

## Findings

We present two overarching themes within this woman’s experience: “vicious” and “virtuous” circles, attempting to make sense of her experience of isolation and lack of wellbeing, and subsequent confidence, engagement and community-building. To maintain anonymity, the participant has been given the name Nala, and further biographical details have been omitted.

### Vicious Circle

#### Early life, pre-pregnancy pressures, and background to sense of privacy

Having escaped the Somali Civil War, Nala spent several years in a transit country before reaching the UK as an adolescent. Some of her family were able to join her, the rest (including her mother) stayed behind. *A*s a teenager, she struggled with her appearance due to a distressing skin condition, without speaking about it with her father, her friends, or her sisters.

I spent hours looking in the mirror hoping it would go away but it wouldn’t.

I didn’t want people to see I was weak because … that’s when people start picking up on you. … so I never showed anyone that it bothered me … I would just go in the toilet at night and look at myself and cry … in the morning I would go to school normal …

My friends … even my sisters … I wouldn’t talk to them about it because they wouldn’t understand … I couldn’t go to my dad to cry about it, we would just go to the skin area of Boots, I think he was more understanding even though he wouldn’t … have a conversation about it.

Despite this, overall Nala appeared to enjoy her UK school life with a circle of friends and the desire to pursue further education, but then a relationship came along.

I got married … [while I was still at school]. I had a relationship … so we decided to get married because in Islam you are not allowed…to have sexual … relations without getting married … My dad, he was quite shocked … but I was like nah I think this is good for me so … my dad … went along with it.

Once married, she juggled schoolwork, a part-time job, and home duties; pulled between Somali, Islamic, and White, British expectations, limiting what she shared with others.

I was doing my A-levels whilst married … that was a bit challenging … I always wanted to do … teaching … [or] midwifery … like coming home, being a wife, going to school but only my closest friends knew, most of the teachers didn’t know.

Nala appreciated her husband’s support for her continuing education, but also felt a cultural expectation to become pregnant quickly. When pregnancy did not come, shame compelled her to keep this secret, however this left her feeling alone.

Once you get married people expect that you get pregnant so quick …

That in itself comes with a little bit of shame because I’m like “oh what’s wrong with me? Why can I not get pregnant?” … and I would pretend and be like “now obviously I’m studying so I don’t want to have kids”, but that wasn’t the case …

If I come out like oh no I can’t get pregnant … not a lot of people would understand.

#### Distressing and isolating experiences of pregnancy

The desire to get pregnant led Nala into a difficult journey with the healthcare system, with her father and husband supporting her journey through fertility treatment financially and practically.

The gynaecologist … was like I’m sorry we cant sent you to the ovulation clinic because you are too young … Me and my husband … went all the way to … this doctor in London … privately. … my dad even gave me the money. We didn’t even have the money at that time …

Nala finally became pregnant after about 2 years. The combination of morning sickness, the loss of her career drive, feeling left behind by friends who had continued to university, and a lack of focus in her daily life left her feeling alone, as if life had lost normality. Time alone, without other focus of activity, intensified negative feelings.

I couldn’t get pregnant until after 2 years when I finished year 13. … I was working, I was going to school and coming home, I had my other duties, I had to do like cook or look after the house or clean or whatever.

… I got pregnant … and wow … everything changed … I had really bad morning sickness … I didn’t want to talk to anybody because I had a massive headache and my mood swings were all over the place … So yeah I would just stay on the sofa.

Differing expectations between Somali and Western cultures/contexts brought internal conflict and increased feelings she didn’t feel able to share, refusing to speak even to her extended family for several months, isolated, without self-confidence.

My husband was working long hours and I would be in the house all by myself … my sister would go to school … and I would have no one … . I would just stay on the sofa for when I needed to throw up…. … I would get angry really quickly.

I think it got me down … I would just get into my own head and thinking about like everything that I lost … All I wanted to do was be on my own … I had no interest to do anything … because at the time I was struggling with the fact that I couldn’t go to uni, with everything that I had sacrificed.

and I remember my husband would call my sister and ask her to … try and get me out of the house but nobody could get me out the house. … my dad would call me and say go to your sisters so she can cook for you and look after you but I didn’t want anyone around.

#### Birthing experience: exhaustion, isolation and disconnect

Nala felt her first birthing experience had a long-lasting, negative effect on her. She felt alone and unprepared for the responsibility of a baby, as a young mother with little exposure to babies, and without the supportiveness that her family took for granted back home, exhausted by the lack of sleep.

You are emotional, scared, you don’t know what to do and the people who are there to support you, you feel like they are not there … .

In the hospital … you don’t get rest … every 20 minutes someone is coming to check you … no one even lets my husband stay during the night which … is the time I needed him the most

Her experience of access to milk supplies in hospital for the birth of her second child (her son) was of racist treatment. With her first child (her daughter), Nala was allowed to access milk supplies freely whenever she wanted, so she assumed this would be the same when she had her second child. However, when she went to get some milk for her son in the hospital, the midwife told her she wasn’t allowed. Nala questioned this but she was told the rules had changed, and she was to get her husband to bring her some milk. She then saw other mums accessing the free milk. Later, when the midwife shift had changed and Nala was making her own milk, the night midwife asked what she was doing and told her that she was allowed to take the milk. Nala was confused and frustrated as she thought the first midwife made certain rules for certain people and different rules for others, in a potentially racist way. She tried to complain formally a year later, but felt this didn’t work.

I had this midwife in charge [who said] I couldn’t take … pre-made milk from the nursery … with my daughter we could just go take it … you will have to get your own powder and make it … sorry the rules changed. … And then the night shift midwife … was like how come you are making your own milk? … there is already ready made one in the nursery. … And the crazy thing was I was seeing people bringing milk from the nursery, and I was like how is this working? … is there rules for certain people and rules for another people, what’s going on?

Why would she tell me I wasn’t allowed to get milk from the nursery when other people could? … and that experience stuck with me …

Maybe she didn’t like me and the fact I was wearing a hijab … That experience stuck with me … a year after, I went to complain, I tried to call but they said I needed to come in. … so I couldn’t even make the complaint that I wanted to make … so what’s the point? … I don’t have time for that … But that stayed with me, that midwife … it just made me so angry, like what was she thinking?

She felt that most hospital staff were not able to support her in the way she needed, which left her feeling alone and disconnected. She attributed this to a lack of training and experience of supporting black and minority ethnic women as they paid limited attention to her, which she took personally, hearing staff talking about her which she found upsetting, judgemental, overwhelming, and discriminatory.

… my daughter [was] … crying all the time, she wants me to pick her up and my whole body was in pain, I was mentally and physically exhausted and … the midwives, they were like “oh just buzz this button if you need help” but every time you buzz … nobody is coming… I just … I broke down

I overheard the conversation and [another midwife] was talking behind my back and … she didn’t understand … … maybe she didn’t like me … because I was black, I don’t know.

The negative birthing experience overall made her reconsider having more children and shaped her experience of early motherhood.

I think the most stressful thing that put me off having kids was the stay in the hospital… you don’t get rest

I couldn’t comprehend going through that again with the hospital and the midwife … I feel like the experience that a lady has with delivery and in the ward shapes her life and stays with her for a long time.

#### Post-natal challenges

Nala described early motherhood after the birth of her first child as a distressing and isolating time when her social confidence diminished, barely interacting with adults outside of her immediate family and avoiding new contacts.

Once you give birth there is postnatal depression and all that … I had a negative experience. Do you know the effect that has on your mind? You overthink stuff? And even when you are in the situation even little things set you off, you are emotional scared you don’t know what to do.

They were the only people I saw … to my sisters house and back, we wouldn’t even go to the park. Because … at that time I lost a lot of confidence … even the thought of making new friends … that was so scary.

Everything that I wanted to do I would be like no I don’t think I can do this … I didn’t even know there was a family centre in [local neighbourhood] … I didn’t even know it existed, that’s how isolated I made myself to be.

… you know the thought of facing new people? That put me off [going out] … I felt like I had nothing to offer.

#### Socio-cultural context

For Nala, emotional reserve, concern over self-image, worrying about appearing vulnerable, and expectations of her as a young married woman contributed to her not speaking about things that were troubling her. This appeared to make sense in a socio-cultural context that included the family experience of migration, separation, and expectation to keep feelings private; cultural experiences (variously Somali and British) of support, expectation, opportunity, and negative treatment, and an apparent conflict or contradiction between cultural expectations of women staying home, with primary responsibility for others, or going out to study or work. We hope this has been illustrated in each of the sub-themes above but have added some additional quotes here.

[My mother] couldn’t come [to the UK], I think she got refused so many times because of her English and things like that.

I felt like the doctors were not understanding, my dad was there so I couldn’t break down and start crying …

A lot of my husband’s friends and they were like oh is your wife pregnant? And that is like a normal thing in our culture you know, for people to ask.

Nala felt her difficult experience of early motherhood was one shared with many Somali women.

Once you have kids your confidence goes really low because you aren’t going out and socialising, working, you know? I think with most Somali ladies here and myself too, when you are not working you are just by yourself. You are with the kids all the time and you almost forget how to socialise with adults.

Perhaps because she was speaking with a white British interviewer while seeming to highlight specifics of Somali culture, noting opportunities, expectations and challenges from living as a Muslim within British culture seemed more taken for granted and internalized.

Somali women, wherever we are in the world, once we have kids we tend to forget about each other and ourselves

When you are weak that’s when people [in school] start picking up on you, and I didn’t want that to happen. So, I never showed anyone that [skin condition] bothered me

I wanted to get into midwifery. Yeah that is what I always wanted to do - it was between teaching and midwifery.

I was struggling with the fact that I couldn’t go to uni, with everything that I had sacrificed.

Cultural differences in experiences of support, connection and isolation were prominent. She saw fears about being spoken about negatively influencing people’s decisions not to share difficult experiences.

At that time … I feel like most of my friends moved on … because like they were in a different like level to me because they were not married or having kids … that was like really isolating.

This is not Africa. In Africa you give birth, your family goes and does the shopping … everything is there. But here, most people don’t have family, you have to be set from the get go.

In the Somali community talking can be hard, there’s a lot of people going through a lot but people don’t always trust each other isn’t it? They don’t want to say anything because this person could tell my business outside

#### Theme summary

Nala’s experience during each of the life phases described in this theme can be seen as a vicious circle. Keeping insecurities and negative life experiences concealed or disconnected from others led to her feeling alone, with reduced confidence and self-worth, and consequently staying home and socializing less, which in turn denied her opportunities for positive interaction and left time to dwell on negative thoughts, a circle/cycle of spiralling distress, lack of confidence, and isolation. This is exemplified by the following quote:

… [a] typical day [when] was I was pregnant … I didn’t want to talk to anybody … my mood swings were all over the place so I would get angry really quickly…I would just stay on the sofa for when I needed to throw up … my husband he was working long hours and I would be in the house all by myself … [he] would call my sister and ask her to call me in the morning and try and get me out of the house but nobody could get me out the house. I remember my dad would call me and say “go to your sister’s so she can cook for you and look after you”, but I didn’t want anyone around.

**Figure 1.**
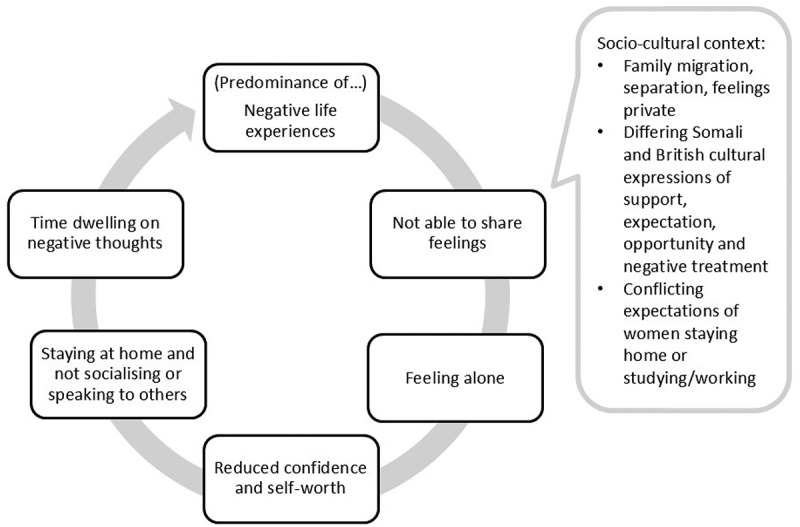
Vicious circle.

The operation of this vicious circle and its socio-cultural context is summarized in Figure 1.

During this difficult time, it seems hard to imagine that Nala’s subsequent experience would be of social connectedness, community, and confidence—we have described this contrasting “virtuous circle” below.

### Virtuous Circle

After her baby was born, Nala gradually began to socialize, slowly breaking out of a vicious circle of low confidence (described above) and, with continuing support and encouragement from family and friends, gradually establishing herself in a “virtuous circle” of growing confidence. Family and friends’ consistent messages to Nala about the importance of getting out and having human contact were rejected many times.

Nobody could get me out the house. I remember my dad would call me and say go to your sisters so she can cook for you and look after you, but I didn’t want anyone around, that’s the thing … my husband would call my sister … I had this one friend from high school … she would call me and ask if I wanted to go out today … At that time, I had no interest to do anything and if you told me to join a group, I would say are you kidding me?

Eventually, experiencing some improvement in her wellbeing and morning sickness from 6 months into the first pregnancy, she had a moment of reflection about the limitations of the life she was leading and what might be possible. Making the first change of recovery, she finally accepted going to her sister’s to be cooked and cared for. After her child’s birth, itself a traumatic experience, she remained depressed and isolated, but the persistent encouragement from her close contacts continued, now with the added direction towards taking her child out to a parent & baby group.

So at 6 months into my pregnancy the sickness started getting better and then I started eating, I start getting well, I was like oh this is not a way to live [depression, isolation] … I started going out [a little] like visiting mostly my sister.

… my sister … would call me and ask if I wanted to come [to the parent & baby group] and I would say no, what’s the point? I don’t want to come.

Giving birth to her second child several years later was a contrasting, and all round more positive experience. She was able to rest when a student midwife offered to look after her newborn to allow Nala to rest. She also appreciated staff who took a comforting and personal approach.

[The midwife] was calming me down, she would tell me everything was going to be okay because I was in so much pain … every time he will cry she will feed him and look after … I was much happier and much calmer because I got sleep. I’m so grateful she did this for me because … that gave me another experience.

After her friend added her voice to the encouragement to attend the local baby group, she finally agreed.

My friend told me about this baby group because she went with her son … Then every time she would tell me about the baby group, about the baby group, about the baby group, so this one time I decided to take my daughter.

The final ingredient that led to regular social connection was Nala’s response to her daughter (now aged two) having separate needs and experience. Watching her daughter’s enjoyment of the opportunity for play and interaction, she experienced a second key moment of reflection, combining guilt about how her isolation might be holding back her child’s development. With this motivation and reinforcement, she continued to attend the baby group. She could now receive the support of others that became so valuable.

Seeing my daughter play there and she had every toy and I don’t know, something came to me … And I started feeling guilty, I started feeling like I was holding my daughter back, am I holding her development back? Because of me wanting to stay in the house? Because she is a child, surely she should be outside. She should be playing with other kids. So I thought I need to try for her, even though I don’t feel like going out and facing other people …

We went again, then I started going regularly and that is how I started coming out of the isolation that I put myself and my daughter into.

Having left the home to attend, one group opened her eyes to the benefits of wider connections.

I had started going out and enjoying the outside so I wanted to fill my time.

I started meeting people in the park.

Her next key steps into a positive cycle of connection, confidence and action came from meeting other women with similar experiences.

I remember one lady who was a young mum, she was Somalian as well … Then she told me about this [Women’s] group … I used to put it off … like it is going to be too much, or too scary … 3 weeks later I decided to go. I went and all the ladies speak Arabic and I am the only Somali lady but … the lady who ran the group … was so nice … and even though they were speaking Arabic I felt connected with them, because they are mothers from ethnic minority.

“Through joining the women’s group, that gave me confidence … through meeting them I wasn’t scared like to meet [other] women … and not thinking so much and putting yourself down … .

I remember I would look forward to the group!… Every Friday my husband would be like, where are you going? I’m going to the group! (laughs) Till now, he knows, its Friday I’m out till 1 o’clock, sometimes 2 o’clock because the group finishes at 12 but we still stay. Nobody wants to leave …

Nala felt that having a purpose, plans and a support network made her second pregnancy more manageable. She compared the difficulties of time alone and a lack of focus during her first pregnancy to her busy, sociable, and directed second pregnancy.

… that [second pregnancy] was different because I couldn’t even be sick because I had … my daughter who was 2 years old and running around … to the point where if I felt like I had to throw up I couldn’t because I couldn’t take the time off! (laughs) … and also I was going out, I was more social than when I was pregnant with my daughter.

In Nala’s experience, communal Somali culture favours advice to manage problems, strengthen relationships, and provide support and empowerment, contributing to a feeling of trust, togetherness and community from getting to know one another and sharing personal issues safely.

All the worries that you got, you have women who have been through it. You can say this is new to me, what do I do? Give me advice.

As Nala gained further self-confidence, she in turn began to encourage, advise, and inspire confidence in others, from her experience, and to step out into a wider range of social spaces.

And every time I chat to people they are in the same situation that I am. … And these ladies they are finding it difficult to express themselves in English and they are coming out and doing something you know? I thought, aright then, I’m not actually so bad, there are things that I can offer.

So I meet people in the park and chat to them like oh how are you? …I’d tell the Somali ladies, there’s this group, it’s literally amazing like you guys have to come. They would listen to me and they would be like alright, alright, nobody comes. And then 3 weeks later, 2 ladies started coming and then 4 weeks later, 3 ladies started coming … 

The relationships established through this group seemed to be contributing very much to a sense of connected and supportive community as highly valued by Somalis.

[At the Women’s Group] we … started laughing, chatting, giving advice and blessing and praying for each other.

A lady who was pregnant and new to the country … we put money together so she can support herself … the ladies brought food … tea … cakes they made. We said may god make it easy for you, your delivery, may you be safe. She was so happy, she hasn’t got any family here, imagine how hard it is … day to day what she needs to do, she calls us.

Giving help as well as receiving it further extended Nala’s sense of purpose and connection, resulting in a sense of being at the heart of community connectedness.

When I’m doing the group, I see I’m making a difference in peoples lives … they push me, they want to do the group because they like It and enjoy it … I just thrive from helping people.

Interviewer: “So, do you feel part of the community now?”

Participant: “Now, yeah … I feel like at the heart of the community (laughs). It’s so strange, when I look back at my life, the difference … now, it’s massive. I have been [here] … 11 years now … before I got married I was just thinking for myself … and now [at the Women’s Group] … I feel like all these women are looking at me, depending on me … I feel like I have my family … my husband, my kids and I have the community, the women’s group.

#### Socio-cultural context

Relevant socio-cultural contexts for this virtuous circle include aspects of family, community, neighbourhood and professional support. Nala experienced a consistent and persistent sense of support from her extended family.

My mum … is a very strong business woman, she supported 7 kids … [in Somalia] the only breadwinner.

I have always been close to my dad … He’s always so supportive, even now. [When not speaking to anyone in the first pregnancy] he would check up on me through my husband.

I had my little sister staying with me at the time who would help me out with whatever I needed.

She highly valued and could rely on a communal Somali culture of mutual support, encouragement and advice, with specific groups especially important at different points.

We always ask advice of each other … All Somali ladies do it.

I have the community, the women’s group.

Places in the local neighbourhood for people to meet also played an important function.

And through that we started coming to the park and I started meeting people.

The lady who runs the [local] network that supports community projects … found a place in [local area] … and then people started coming.

In her second pregnancy, Nala had a really positive experience of support from the midwives, and was able to access a parent & baby group at a local family centre.

I had two really good midwives with my son … they were amazing.

My older sister … had this baby group at a family centre she used to take her son to.

#### Theme summary

Nala’s experience during this phase of her life can be seen as a virtuous circle of connection, confidence and action. Making social connections with other women with similar experiences, and distracted by this from negative, self-deprecating thoughts, she could receive support, encouragement and advice she could trust. Responding to this by doing more of the things that mattered to her brought a sense of fulfilment as well as increasing confidence and motivation, both to do more, and to make more social contact that could develop the connection and trust further. The circle was further extended by Nala offering support, advice and assistance to others, bringing greater benefit and further developing her role and sense of identity within the community.

**Figure 2.**
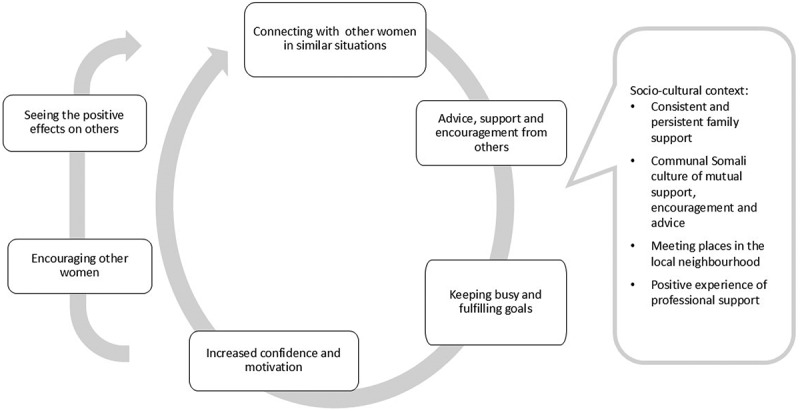
Virtuous circle.

The following diagram (Figure 2) attempts to portray this circle, as experienced by Nala.

## Discussion

From Interpretative Phenomenological Analysis of this single case study of a Somali woman’s experience of pregnancy and early motherhood in an urban UK setting, we describe a shift from a “vicious circle” of lack of wellbeing and isolation to a “virtuous circle” of gaining confidence and engaging with others that places her at the heart of a committed and caring community.

Presenting the account of a single participant, this study makes no attempt to represent Somali women in Bristol, let alone the range of Somali experience in the UK; we do not argue that these are the only conclusions that could be drawn from this study. However, we have attempted to present a coherent and persuasive interpretation of this woman’s experience, which we believe offers useful points of theoretical generalization (Radley & Chamberlain, 2012). These include social contexts for experience and meaning, which may be common to other migrants from “communal” or “collective” societies to urban, western environments, about feedback loops connecting context, meaning and behaviour in the form of “vicious” and “virtuous” circles of social isolation and connection.

### Social context of experience and meaning

Nala’s story illustrates the complex interweaving of personal, relational, and cultural meanings and behaviours that can contribute to strength and vulnerability at the entry to adulthood, marriage, and pregnancy. Inability, in her socio-cultural context, to share feelings appeared to contribute to social withdrawal, and missing out on opportunities for positive interaction that mutually reinforced the “vicious circle” during early married life, pregnancy, birth and early motherhood. The challenges and assets we describe are consistent with those from other studies (Allport et al., 2019; Bentley et al., 2020; Gele et al., 2020; Heslehurst et al., 2018; Lightfoot et al., 2016; Mugadza et al., 2019; Osman et al., 2016), reviewed richly by Pangas et al. (2019), and Kingsbury & Chatfield (2019), with stress, stigma, isolation and the importance of supportive social networks prominent findings for migrant women during pregnancy (Benza & Liamputtong, 2014; Winter et al., 2023); these experiences pose important challenges for maternity and post-natal services (Fair et al., 2020; Heslehurst et al., 2018; Small et al., 2008).

Nala’s description of oral communication, including advice from trusted informants, as highly valued in Somali culture, has been emphasized in other reports (Ahmed, 2002; Markussen, 2020). At the same time, migrant women’s experience of advice, so valued in relation to the uncertainties posed by pregnancy, can be complex—with different sources of advice contrasting or contradictory (Benza & Liamputtong, 2014), or poorly culturally attuned, with consequent dissatisfaction with healthcare advice (Betancourt et al., 2015; Njenga, 2019; Pavlish et al., 2010). Our findings are consistent with this, underlining the importance of advice that is attuned and aligned to individual, social and cultural context.

More generally, her experiences of conflicting or contradictory cultural expectations, and their effects, link to literature about challenges for migrant women negotiating life and access to services during pregnancy and motherhood (Hodgkinson et al., 2014; Pangas et al., 2019), and to powerful individual and communal effects of inclusion or exclusion that can come from socio-cultural experiences and expressions of support, expectation, privacy, shame, connection and trust as well as inequalities in power and resources (Bakuri et al., 2020; Byrow et al., 2020; Eklöf et al., 2016; Selman et al., 2018; Sobral, 2019; Ussher et al., 2017). For migrant women, the loss of their former social network together with new, unfamiliar, potentially contradictory expectations from living within a new culture (Baghdasaryan et al., 2021; Osman et al., 2016), can serve as a “double burden” contributing to experiences of loneliness, isolation or social withdrawal (Jopling & Sserwanja, 2016; Lee et al., 2019).

Our findings highlight the potential importance of interpersonal connection and support for women in Somali and other migrant communities, and accessibility of physical and social resources and assets (Fletcher & Sarkar, 2013; Ungar & Theron, 2020) that may facilitate this. Given the increasing understanding of the importance of pregnancy and early life experiences also for early child development and wellbeing through epigenetic and other mechanisms (Masten et al., 2021), these findings are also important for future generations.

### Alignment and relational/cultural processes of resilience

We describe the important shift Nala made, between the “vicious circle” of low self-esteem and isolation and the “virtuous circle” of gaining confidence and engaging with others, as having required several aspects of Nala’s experience, both external and internal, to be aligned, in the sense that the continuing support and encouragement from family and friends, key moments of self-reflection and change, and appreciation of her daughter’s separate needs and experience, all pointed in a similar direction—towards more social experience. The small first steps towards change were then reinforced by positive feedback from meeting others.

**Figure 3.**
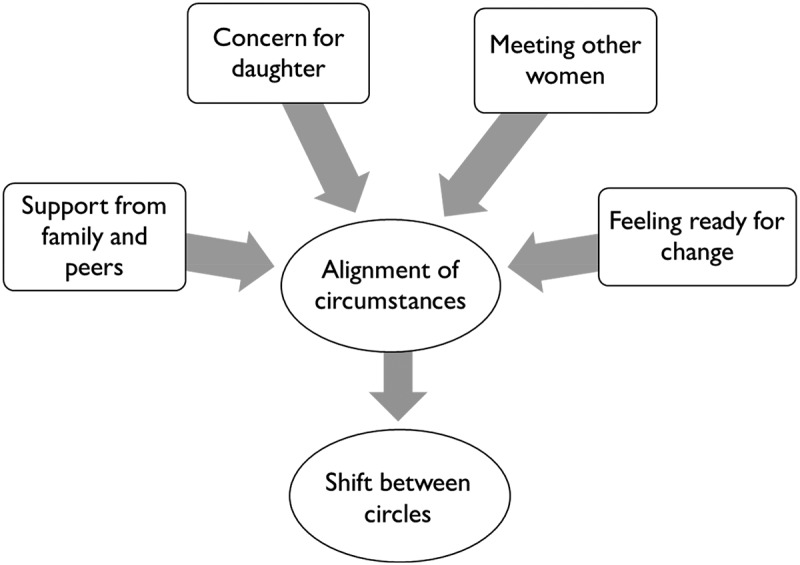
Alignment of circumstances.

The following diagram (Figure 3) attempts to portray the circumstances contributing to a sense of alignment, as experienced by Nala that allowed shift between vicious and virtuous circles.

The experience of “alignment” in a pro-social direction at the crucial shifting point between vicious and virtuous circles offers a further potential point of theoretical generalization. The concept of alignment has become popular in organizational, educational, communication, policy, community development and public health theory (Kagan & Kauerz, 2007; Nilson, 2018; Pearce & Pearce, 2004; Risser et al., 2011; Von Thiele Schwarz et al., 2013; Academy of Medical Sciences, 2016). This draws on ecological ideas of mutual “fit” or “coupling” between organism and environment as applied to human systems (Atkins et al., 2016; Hawe et al., 2009) and considered as directional processes or vectors (Shah, 0000). It has been used in relation to ideas of resilience in urban policy, public health and post-conflict contexts (Croese et al., 2020; Plough et al., 2013; Zraly & Nyirazinyoye, 2010).

(Masten et al. 2018; Masten et al., 2021) note that parallel, aligned resilience processes can be observed in systems at multiple levels. We observed a number of key relational processes within our findings which seemed to help understand the experiences and outcomes that Nala described—these can be listed as follows (Table I):

**Table I.** Key relational processes.

Relational process	Illustrative quote
Negotiation of feelings, activities, responsibilities, and meaning within close relationships	*“My friend … every time she would tell me about the baby group, about the baby group, about the baby group, so this one time I decided to take my daughter.”*
Identity (re-)negotiated and validated interactionally in social networks, and through choice and success of activities	*“I feel … at the heart of the community (laughs) … I feel like all these women are looking at me, depending on me.”*
Differing or contradictory cultural expectations posing dilemmas and challenges, amplified at times of transition such as pregnancy, childbirth and early motherhood	*“In my first pregnancy my husband was working long hours … my sister would go to school … and I would have no one and I think [that] got me down … I would just get into my own head and thinking about like everything that I lost … friends … I couldn’t go to uni, with everything that I had sacrificed.”*
Physical and social environments supporting community connectedness	*“The lady who runs the [local] network that supports community projects … found a place in [local area] … and then people started coming.”*
Experience of professionals influenced by (dis)advantage, community networks and culture.	*“I had this midwife in charge [who said] I couldn’t take … pre-made milk from the nursery … and I was like how is this working? … is there rules for certain people and rules for another people, what’s going on?”*

The identification of processes such as these is consistent with advances in research on resilience focused on social contexts, such as family and cultural systems (Masten & Cicchetti, 2016). In this complex interwoven social context, the experience of “alignment” appeared to simplify Nala’s choices and release her from the vicious circle she was experiencing, to permit the transformation that she described from a place of low self-esteem and social withdrawal, to providing a rich contribution to the community.

## Conclusions

In this study of a single Somali woman’s experience of pregnancy and early motherhood in an urban UK setting, we describe a journey from a “vicious circle” of lack of wellbeing and isolation to a “virtuous circle” of confidence, engagement and community-building, with change becoming possible when her experience of social support and feedback all seemed aligned towards more social contact. Practitioners and researchers exploring processes of change and resilience may wish to attend to the directionality of change that is offered and experienced, and its fit with other psychosocial contexts. For example, when listening to the thoughts and feelings of individuals about choices they may have, practitioners (as well as family and friends!) may find it helpful to explore how aspects of their social relationships may bring congruities and/or dissonances in the “direction of travel” towards action. This may then help them to align their subsequent contribution to individuals’ lives in a more useful way, potentially assisting the transitions of identity, responsibility, health/wellbeing, and activity that take place in pregnancy and early motherhood.

Given the importance of social isolation and connection for migrants described in the wider literature as well as prominently in this study, we would urge authorities and commissioners to increase support for community activities and organizations that can foster positive social networks and peer support.

## Supplementary Material

Biographical note.docx

## Note

1. The ethical approval also required participants not to have a current pregnancy.

## Appendix Semi-structured Interview Schedule

**Table:**

Category	Questions [prompts in square brackets]
Daily routine	Please take me through a typical day for you during pregnancy: [Time/activity/place/person/ … When/What/Where/Who. How did the daily routine vary from day to day during the pregnancy? [What things happened that needed to respond to/adjust your life for? Where did you go out to? [Are there certain places you found comforting/particularly liked?] Who would you see throughout the day? [Did you see family? Are many of your family in Bristol? Where are other people in your family? What contact do you have with them? Were there days you might not see anyone? What was that like? When you were out, who do you meet (planned/unplanned)? What was that like? How did the people you saw/didn’t see make a difference for your pregnancy?] What made for positives or negatives experiences in pregnancy? [What mattered for this? What was your favourite/least favourite part of the day? What made your day easier/harder? Who did you like/not like seeing?]
Support network	Who helped and supported you during your pregnancy and once the baby was born? [Friends? Husband? Family? Other people you see … every day/every week/at particular times?] How much did you feel part of a community here? [What would that community be? When did you feel most part of it? What helped/would help that community to be good for pregnant women and new mothers?]
Wellbeing & Stress	Tell me about a time you felt (really positive/really stressed) during your pregnancy/early motherhood [What was it about that time that made it (really positive/really stressful)? What did you do? What did you/could we learn from that experience?] Have you seen other women having a particularly positive/particularly stressful experience during pregnancy? [How did that show? What do you think contributed to that? What could we learn from those experiences?]
Baby arrival	How did you prepare for raising your child? How was it when the baby actually came?
Final checks	Are there some areas we haven’t touched on? Would you like to talk about anything else? Should we have asked anything else? I feel like X was important for you—would you like to talk some more about this? In your opinion, what are the main things that help (and/or challenge?) a Somali woman in Bristol feel socially connected and included during pregnancy? What could people do to make things better? Check if demographic questions answered (*if they wish*) [Length of time in UK/Bristol? Who live with? Number of previous pregnancies/children? When most recent baby born? Family members in Bristol/elsewhere?

## References

[cit0001] Academy_of_medical_sciences. (2016). *Improving the health of the public by 2040. Optimising the research environment for a healthier, fairer future.* Retrieved Accessed October 23, 2024, from https://acmedsci.ac.uk/file-download/41399-5807581429f81.pdf

[cit0002] Ahmed, A. J. (2002). The Somali oral tradition and the role of storytelling in Somalia. Retrieved October 23, 2024, from https://somali.wdfiles.com/local-files/readings/AhmedMHC.pdf

[cit0003] Allport, T., Mace, J., Farah, F., Yusuf, F., Mahdjoubi, L., & Redwood, S. (2019). ‘Like a life in a cage’: Understanding child play and social interaction in Somali refugee families in the UK. *Health & Place*, 56, 191–15. 10.1016/j.healthplace.2019.01.019 30825824

[cit0004] Atkins, M. S., Rusch, D., Mehta, T. G., & Lakind, D. (2016). Future directions for dissemination and implementation science: Aligning ecological theory and public health to close the research to practice gap. *Journal of Clinical Child & Adolescent Psychology*, 45(2), 215–226. 10.1080/15374416.2015.1050724 26155972 PMC4706825

[cit0005] Baghdasaryan, Z., Lampa, E., & Osman, F. (2021). ‘Let us understand each other and work together in the child’s best interest’ – exploring the narratives of newly arrived refugee parents in Sweden. *International Journal of Intercultural Relations*, 81, 226–235. 10.1016/j.ijintrel.2021.02.004

[cit0006] Bains, S., Skråning, S., Sundby, J., Vangen, S., Sørbye, I. K., & Lindskog, B. V. (2021). Challenges and barriers to optimal maternity care for recently migrated women-a mixed-method study in Norway. *BMC Pregnancy and Childbirth*, 21(1), 1–14. 10.1186/s12884-021-04131-7 34620114 PMC8495671

[cit0007] Bakuri, A., Spronk, R., Dijk, V. A. N., & R. (2020). Labour of love: Secrecy and kinship among Ghanaian-Dutch and Somali-Dutch in the Netherlands. *Ethnography*, 21(3), 394–412. 10.1177/1466138120938808

[cit0008] Bentley, J. A., Mohamed, F., Feeny, N., Ahmed, L. B., Musa, K., Tubeec, A. M., Angula, D., Egeh, M. H., & Zoellner, L. (2020). Local to global: Somali perspectives on faith, community, and resilience in response to COVID-19. *Psychological Trauma: Theory, Research, Practice, & Policy*, 12(S1), S261–263. 10.1037/tra0000854

[cit0009] Benza, S., & Liamputtong, P. (2014). Pregnancy, childbirth and motherhood: A meta-synthesis of the lived experiences of immigrant women. *Midwifery*, 30(6), 575–584. 10.1016/j.midw.2014.03.005 24690130

[cit0010] Betancourt, T. S., Abdi, S., Ito, B. S., Lilienthal, G. M., Agalab, N., & Ellis, H. (2015). We left one war and came to another: Resource loss, acculturative stress, and caregiver–child relationships in Somali refugee families. *Cultural Diversity & Ethnic Minority Psychology*, 21(1), 114–125. 10.1037/a0037538 25090142 PMC4315611

[cit0011] BonmatÍ-Tomás, A., Malagón-Aguilera, M. D. C., Bosch-Farré, C., Gelabert-Vilella, S., Juvinyà-Canal, D., & Garcia Gil, M. D. M. (2016). Reducing health inequities affecting immigrant women: A qualitative study of their available assets. *Globalization and Health*, 12(1), 1–10. 10.1186/s12992-016-0174-8 27388538 PMC4936252

[cit0012] Braun, V., & Clarke, V. (2006). Using thematic analysis in psychology. *Qualitative Research in Psychology*, 3(2), 77–101. 10.1191/1478088706qp063oa

[cit0013] Bronfenbrenner, U. (1986). Ecology of the family as a context for human development: Research perspectives. *Developmental Psychology*, 22(6), 723–742. 10.1037/0012-1649.22.6.723

[cit0014] Byrow, Y., Pajak, R., Specker, P., & Nickerson, A. (2020). Perceptions of mental health and perceived barriers to mental health help-seeking amongst refugees: A systematic review. *Clinical Psychology Review*, 75, 101812. 10.1016/j.cpr.2019.101812 31901882

[cit0015] Cao-Lei, L., De rooij, S., King, S., Matthews, S., Metz, G., Roseboom, T., & Szyf, M. (2020). Prenatal stress and epigenetics. *Neuroscience & Biobehavioral Reviews*, 117, 198–210. 10.1016/j.neubiorev.2017.05.016 28528960

[cit0016] Clark, C. L., Glavin, K., Missal, B. E., & Sæteren, B. (2018). Is there a common experience? Somali new mothers’ childbirth experiences in Norway and the United States. *Public Health Nursing*, 35(3), 184–191. 10.1111/phn.12399 29566259

[cit0017] Cooperrider, D., & Whitney, D. D. (2005). *Appreciative inquiry: A positive revolution in change*. Berrett-Koehler Publishers. ISBN 160509692X.

[cit0018] Croese, S., Green, C., & Morgan, G. (2020). Localizing the sustainable development goals through the lens of urban resilience: Lessons and learnings from 100 resilient cities and Cape Town. *Sustainability*, 12(2), 1–16. 10.3390/su1202055035136666

[cit0019] Degrie, L., Gastmans, C., Mahieu, L., Casterlé, D. D., Denier, B, Y., & Denier, Y. (2017). How do ethnic minority patients experience the intercultural care encounter in hospitals? A systematic review of qualitative research. *BMC Medical Ethics*, 18(1), 1–17. 10.1186/s12910-016-0163-8 28103849 PMC5244561

[cit0020] Eatough, V., & Smith, J. (2006). I was like a wild wild person: Understanding feelings of anger using interpretative phenomenological analysis. *British Journal of Psychology*, 97(4), 483–498. 10.1348/000712606X97831 17018185

[cit0021] Eklöf, N., Abdulkarim, H., Hupli, M., & Leino-Kilpi, H. (2016). Somali asylum seekers’ perceptions of privacy in healthcare. *Nursing Ethics*, 23(5), 535–546. 10.1177/0969733015574927 25870175

[cit0022] Evason, S. (2019). *Somali culture: Core concepts*. Cultural Atlas. https://culturalatlas.sbs.com.au/somali-culture/somali-culture-core-concepts accessed:5/4/2024

[cit0023] Fair, F., Raben, L., Watson, H., Vivilaki, V., Van den Muijsenbergh, M., Soltani, H. & Team, O. (2020). Migrant women’s experiences of pregnancy, childbirth and maternity care in European countries: A systematic review. *Public Library of Science ONE*, 15(2), e0228378. 10.1371/journal.pone.0228378 32045416 PMC7012401

[cit0024] Finlay, L. (2002). Negotiating the swamp: The opportunity and challenge of reflexivity in research practice. *Qualitative Research*, 2(2), 209–230. 10.1177/146879410200200205

[cit0025] Fletcher, D., & Sarkar, M. (2013). Psychological resilience. *European Psychologist*, 18(1), 12–23. 10.1027/1016-9040/a000124

[cit0026] Flyvbjerg, B. (2006). Five misunderstandings about case-study research. *Qualitative Inquiry*, 12(2), 219–245. 10.1177/1077800405284363

[cit0027] Gele, A. A., Musse, F. K., Shrestha, M., Qureshi, S., & Enuameh, Y. A. K. (2020). Barriers and facilitators to contraceptive use among Somali immigrant women in Oslo: A qualitative study. *Public Library of Science ONE*, 15(3), e0229916. 10.1371/journal.pone.0229916 32155181 PMC7064199

[cit0028] Giscombe, T., Hui, A., & Stickley, T. (2020). Perinatal mental health amongst refugee and asylum-seeking women in the UK. *Mental Health Review Journal*, 25(3), 241–253. 10.1108/MHRJ-01-2020-0008

[cit0029] Glasscoe, C., & Smith, J. A. (2008). Through a mother’s lens: A qualitative analysis reveals how temporal experience shifts when a boy born preterm has cystic fibrosis. *Clinical Child Psychology and Psychiatry*, 13(4), 609–626. 10.1177/1359104508096772 18927144

[cit0030] Hawe, P., Shiell, A., & Riley, T. (2009). Theorising interventions as events in systems. *American Journal of Community Psychology*, 43(3–4), 267–276. 10.1007/s10464-009-9229-9 19390961

[cit0031] Heslehurst, N., Brown, H., Pemu, A., Coleman, H., & Rankin, J. (2018). Perinatal health outcomes and care among asylum seekers and refugees: A systematic review of systematic reviews. *BMC Medicine*, 16(1), 1–25. 10.1186/s12916-018-1064-0 PMC5996508 29890984

[cit0032] Hodgkinson, E. L., Smith, D. M., & Wittkowski, A. (2014). Women’s experiences of their pregnancy and postpartum body image: A systematic review and meta-synthesis. *BMC Pregnancy and Childbirth*, 14(1), 1–11. 10.1186/1471-2393-14-330 25248649 PMC4261580

[cit0033] Jacoby, S. D., Lucarelli, M., Musse, F., Krishnamurthy, A., & Salyers, V. (2015). A mixed-methods study of immigrant Somali women’s health literacy and perinatal experiences in Maine. *Journal of Midwifery & Women’s Health*, 60(5), 593–603. 10.1111/jmwh.12332 26461193

[cit0034] Jopling, K., & Sserwanja, I. (2016). Loneliness across the life course: Arapid review of the evidence. Gulbenkian Foundation. Retrieved October 23, 2024, from https://content.gulbenkian.pt/wp-content/uploads/sites/18/2016/07/01175346/27-07-16-Loneliness-Across-the-Life-Course-Full-Report.pdf

[cit0035] Kagan, S. L., & Kauerz, K. (2007). Reaching for the whole: Integration and alignment in early education policy. In R. C. Pianta & M. J. C, & K. L. Snow (Eds.), *School readiness and the transition to kindergarten in the era of accountability* (pp. 11–30). Paul H Brookes Publishing

[cit0036] Kelly, S. E., Bourgeault, I., & Dingwall, R. (2010). Qualitative interviewing techniques and styles. In R. Dingwall, I. Bourgeault, & R. DeVries (Eds.), *The SAGE Handbook of Qualitative Methods in Health Research* (pp. 307–326). 10.4135/9781446268247.N17

[cit0037] Kingsbury, D. M., & Chatfield, S. L. (2019). A qualitative metasynthesis of published research exploring the pregnancy and resettlement experience among refugee women. *The Qualitative Report*, 24, 242–257. 10.46743/2160-3715/2019.3750

[cit0038] Lee, K., Vasileiou, K., & Barnett, J. (2019). ‘Lonely within the mother’: An exploratory study of first-time mothers’ experiences of loneliness. *Journal of Health Psychology*, 24(10), 1334–1344. 10.1177/1359105317723451 28795604

[cit0039] Lewis, J., & Ritchie, J. (2003). Generalising from qualitative research. In J. Ritchie & J. Lewis (Eds.), *Qualitative research practice: A guide for social science students and researchers* (pp. 263–286). Sage.

[cit0040] Lightfoot, E., Blevins, J., Lum, T., & Dube, A. (2016). Cultural health assets of Somali and Oromo refugees and immigrants in Minnesota: Findings from a community-based participatory research project. *Journal of Health Care for the Poor and Underserved*, 27(1), 252–260. 10.1353/hpu.2016.0023 27763468

[cit0041] Mack, N., Woodsong, C., Macqueen, K. M., Guest, G., & Namey, E. (2005). *Qualitative research methods: A data collectors field guide.* Family Health International. Retrieved December 12, 2024, from https://www.fhi360.org/wp-content/uploads/2024/01/Qualitative-Research-Methods-A-Data-Collectors-Field-Guide.pdf.

[cit0042] Madeira, A. D., Rangen, C. M., & Avery, M. D. (2019). Design and implementation of a group prenatal care model for Somali women at a low-resource health clinic. *Nursing for Women’s Health*, 23(3), 224–233. 10.1016/j.nwh.2019.03.007 31077639

[cit0043] Madigan, S., Oatley, H., Racine, N., Fearon, R. P., Schumacher, L., Akbari, E., Cooke, J. E., & Tarabulsy, G. M. (2018). A meta-analysis of maternal prenatal depression and anxiety on child socioemotional development. *Journal of the American Academy of Child and Adolescent Psychiatry*, 57(9), 645–657. e8. 10.1016/j.jaac.2018.06.012 30196868

[cit0044] Markussen, M. K. G. (2020). ‘Nobody comes to Baba for advice’: Negotiating ageing masculinities in the Somali diaspora. *Journal of Ethnic & Migration Studies*, 46(7), 1442–1459. 10.1080/1369183X.2018.1496817

[cit0045] Masten, A. S. (2001). Ordinary magic: Resilience processes in development. *The American Psychologist*, 56(3), 227–238. 10.1037/0003-066X.56.3.227 11315249

[cit0046] Masten, A. S. (2018). Resilience theory and research on children and families: Past, present, and promise. *Journal of Family Theory & Review*, 10(1), 12–31. 10.1111/jftr.12255

[cit0047] Masten, A. S., & Cicchetti, D. (2016). Resilience in development: Progress and transformation. In: D. Cicchetti (Ed.), *Developmental Psychopathology* (pp. 271–333). 10.1002/9781119125556.devpsy406

[cit0048] Masten, A. S., Lucke, C. M., Nelson, K. M., & Stallworthy, I. C. (2021). Resilience in development and psychopathology: Multisystem perspectives. *Annual Review of Clinical Psychology*, 17(1), 521–549. 10.1146/annurev-clinpsy-081219-120307 33534615

[cit0049] Mugadza, H. T., Mujeyi, B., Stout, B., Wali, N., & Renzaho, A. (2019). Childrearing practices among sub-Saharan African migrants in Australia: A systematic review. *Journal of Child & Family Studies*, 28(11), 2927–2941. 10.1007/s10826-019-01463-z

[cit0050] Nilson, C. (2018). Community safety and well-being: Concept, practice, and alignment (LEPH2018). *Journal of Community Safety and Well-Being*, 3(3), 96–104. 10.35502/jcswb.81

[cit0051] Njenga, A. N. (2019). *Perinatal cultural beliefs of Somali refugee women* [Doctoral dissertation]. The University of Utah. https://www.proquest.com/docview/2498537684?pq-origsite=gscholar&fromopenview=true&sourcetype=Dissertations%20&%20Theses accessed:23/10/2024

[cit0052] Noon, E. J. (2018). Interpretive phenomenological analysis: An appropriate methodology for educational research. *Journal of Perspectives in Applied Academic Practice*, 6(1), 75–83. 10.14297/jpaap.v6i1.304

[cit0053] O'Dougherty Wright, M., Masten, A. S., & Narayan, A. J. (2013). Resilience processes in development: Four waves of research on positive adaptation in the context of adversity. In S. Goldstein, & R. Brooks (Eds.), *Handbook of resilience in children* (pp. 15–37). Springer. 10.1007/978-1-4614-3661-4_2

[cit0054] Office_for_National_Statistics. (2023). Somali populations, England and Wales: Census, 2021: Exploring the education, employment, health, housing, language and country of birth of residents who identified as Somali in England and Wales using census 2021 data. Office_for_National_Statistics. Retrieved April 5, 2024, from https://www.ons.gov.uk/peoplepopulationandcommunity/culturalidentity/ethnicity/articles/somalipopulationsenglandandwales/census2021

[cit0055] Oommen, H., Esse, L., Sajer, S., & Lukasse, M. (2024). Somali women’s perceptions and experiences of pain and pain relief during childbirth in Norway: A qualitativestudy. *European Journal of Midwifery*, 8(February), 1–8. 10.18332/ejm/176034 PMC10845056 38323166

[cit0056] Osman, F., KLINGBERG-ALLVIN, M., Flacking, R., & Schön, U.-K. (2016). Parenthood in transition – Somali-born parents’ experiences of and needs for parenting support programmes. *BMC International Health and Human Rights*, 16(1), 1–11. 10.1186/s12914-016-0082-2 26883321 PMC4754847

[cit0057] Pangas, J., Ogunsiji, O., Elmir, R., Raman, S., Liamputtong, P., Burns, E., Dahlen, H. G., & Schmied, V. (2019). Refugee women’s experiences negotiating motherhood and maternity care in a new country: A meta-ethnographic review. *International Journal of Nursing Studies*, 90, 31–45. 10.1016/j.ijnurstu.2018.10.005 30583266

[cit0058] Pavlish, C. L., Noor, S., & Brandt, J. (2010). Somali immigrant women and the American health care system: Discordant beliefs, divergent expectations, and silent worries. *Social Science & Medicine*, 71(2), 353–361. 10.1016/j.socscimed.2010.04.010 20494500 PMC2893335

[cit0059] Pearce, W. B., & Pearce, K. A. (2004). Taking a communication perspective on dialogue. In R. ANDERSON & L. A. B, K. N. CISSNA (Eds.), *Dialogue: Theorizing difference in communication studies* (pp. 39–56). Sage.

[cit0060] Peyton, T., & Wisniewski, P. (2019). Improving a design space: Pregnancy as a collaborative information and social support ecology. In: K. ARAI & R. BHATIA (Eds. Future of Information and Communication Conference (FICC) Proceedings, Springer. 10.1007/978-3-030-12388-8_36 accessed:23/10/2024

[cit0061] Pietkiewicz, I., & Smith, J. A. (2014). A practical guide to using interpretative phenomenological analysis in qualitative research psychology. *Psychological Journal*, 20(1), 7–14. 10.14691/CPPJ.20.1.7

[cit0062] Plough, A., Fielding, J. E., Chandra, A., Williams, M., Eisenman, D., Wells, K. B., Law, G. Y., Fogleman, S., & Magaña, A. (2013). Building community disaster resilience: Perspectives from a large urban county department of public health. *American Journal of Public Health*, 103(7), 1190–1197. 10.2105/AJPH.2013.301268 23678937 PMC3682619

[cit0063] Radley, A., & Chamberlain, K. (2012). The study of the case: Conceptualising case study research. *Journal of Community & Applied Social Psychology*, 22(5), 390–399. 10.1002/casp.1106

[cit0064] Risser, M., O’Neill, M., & Cain, T. (2011). *Achieving policy alignment: A cross jurisdictional study.* Institute on Governance.

[cit0065] Rogers, A., Obst, S., Teague, S. J., Rossen, L., Spry, E. A., Macdonald, J. A., Sunderland, M., Olsson, C. A., Youssef, G., & Hutchinson, D. (2020). Association between maternal perinatal depression and anxiety and child and adolescent development: A meta-analysis. *JAMA Pediatrics*, 174(11), 1082–1092. 10.1001/jamapediatrics.2020.2910 32926075 PMC7490743

[cit0066] Selman, L. E., Fox, F., Aabe, N., Turner, K., Rai, D., & Redwood, S. (2018). ‘You are labelled by your children’s disability’ – A community-based, participatory study of stigma among Somali parents of children with autism living in the United Kingdom. *Ethnicity & Health*, 23(7), 781–796. 10.1080/13557858.2017.1294663 28277014

[cit0067] Shah, D. Aligning vectors: How to scale a business. #INBOUND17, 2017. https://www.youtube.com/watch?v=i0yqJa48ebs accessed:23/10/2024

[cit0068] Sharma, E., Tseng, P.-C., Harden, A., Li, L., & Puthussery, S. (2023). Ethnic minority women’s experiences of accessing antenatal care in high income European countries: A systematic review. *BMC Health Services Research*, 23(1), 612. 10.1186/s12913-023-09536-y 37301860 PMC10256965

[cit0069] Small, R., Gagnon, A., Gissler, M., Zeitlin, J., Bennis, M., Glazier, R. H., Haelterman, E., Martens, G., Mcdermott, S., Urquia, M., & Vangen, S. (2008). Somali women and their pregnancy outcomes postmigration: Data from six receiving countries. *BJOG: An International Journal of Obstetrics & Gynaecology*, 115(13), 1630–1640. 10.1111/j.1471-0528.2008.01942.x 19035939 PMC2659389

[cit0070] Smith, J. A. (1995). Semi-structured interviewing and qualitative analysis. In J. A. Smith, R. Harre, & L. Van Langenhove (Eds.), *Rethinking Methods in Psychology* (pp. 8–26).

[cit0071] Smith, J. A. (1996). Beyond the divide between cognition and discourse: Using interpretative phenomenological analysis in health psychology. *Psychology & Health*, 11(2), 261–271. 10.1080/08870449608400256

[cit0072] Smith, J. A. (2004). Reflecting on the development of interpretative phenomenological analysis and its contribution to qualitative research in psychology. *Qualitative Research in Psychology*, 1, 39–54.

[cit0073] Smith, J. A., Jarman, M., & Osborn, M. (1999a). Doing interpretative phenomenological analysis. In M. Murray, & K. Chamberlain (Eds.), *Qualitative Health Psychology: Theories and Methods* (pp. 218–230). Sage.

[cit0074] Smith, J. A., Larkin, M., & Flowers, P. (2021). *Interpretative phenomenological analysis: Theory, method and research*. Sage. ISBN 9781529753790.

[cit0075] Sobral, A. (2019). ‘My body is burning with the shame of not belonging’: Gender, violence and shame in diasporic Somali women’s writings. *European Journal of English Studies*, 23(3), 326–339. 10.1080/13825577.2019.1655235

[cit0076] Staneva, A. A., Bogossian, F., & Wittkowski, A. (2015). The experience of psychological distress, depression, and anxiety during pregnancy: A meta-synthesis of qualitative research. *Midwifery*, 31(6), 563–573. 10.1016/j.midw.2015.03.015 25912511

[cit0077] Ungar, M., & Theron, L. (2020). Resilience and mental health: How multisystemic processes contribute to positive outcomes. *Lancet Psychiatry*, 7(5), 441–448. 10.1016/S2215-0366(19)30434-1 31806473

[cit0078] UNHCR. (2024. *UNHCR Country Information: Somalia* [Online]. *Organisation website: United nations high commission for refugees*. https://www.unhcr.org/uk/somalia.html Accessed 23/10/2024.

[cit0079] Ussher, J. M., Perz, J., Metusela, C., Hawkey, A. J., Morrow, M., Narchal, R., & Estoesta, J. (2017). Negotiating discourses of shame, secrecy, and silence: Migrant and refugee women’s experiences of sexual embodiment. *Archives of Sexual Behavior*, 46(7), 1901–1921. 10.1007/s10508-016-0898-9 28083724 PMC5547186

[cit0080] Viswasam, K., Eslick, G. D., & Starcevic, V. (2019). Prevalence, onset and course of anxiety disorders during pregnancy: A systematic review and meta analysis. *Journal of Affective Disorders*, 255, 27–40. 10.1016/j.jad.2019.05.016 31129461

[cit0081] Von Thiele Schwarz, U. & Hasson, H. (2013). Alignment for achieving a healthy organization. In G. BAUER & J. GREGOR (Eds.), *Salutogenic organizations and change* (pp. 107–125). Springer.

[cit0082] Wallis, E. (2018). *Young, Muslim and Black: The struggle of integration for Somalis in the UK* [Online]. InfoMigrants. Available: https://www.infomigrants.net/en/webdoc/139/young-muslim-and-black-the-struggle-of-integration-for-somalis-in-the-uk Accessed 5/4/2024.

[cit0083] Wallmo, S., Allgurin, K., & BERTERÖ, C. (2020). The lived experience among Somali women of giving birth in Sweden: An interpretive phenomenological study. *BMC Pregnancy and Childbirth*, 20(1), 1–12. 10.1186/s12884-020-02933-9 PMC7193409 32357845

[cit0084] Winter, A. K., Due, C., & Ziersch, A. (2023). Wellbeing outcomes and risk and protective factors for parents with migrant and refugee backgrounds from the Middle East in the First 1000 Days: A systematic review. *Journal of Immigrant & Minority Health*, 26(2), 1–14. 10.1007/s10903-023-01510-4 37410193 PMC10937786

[cit0085] Woody, C., Ferrari, A., Siskind, D., Whiteford, H., & Harris, M. (2017). A systematic review and meta-regression of the prevalence and incidence of perinatal depression. *Journal of Affective Disorders*, 219, 86–92. 10.1016/j.jad.2017.05.003 28531848

[cit0086] Zraly, M., & Nyirazinyoye, L. (2010). Don’t let the suffering make you fade away: An ethnographic study of resilience among survivors of genocide-rape in southern Rwanda. *Social Science & Medicine*, 70(10), 1656–1664. 10.1016/j.socscimed.2010.01.017 20223570

