# Supplementary material for: ‘At the heart of the community’ – a Somali woman’s experience of ‘alignment’ of support to escape social isolation in pregnancy and early motherhood
Source: Int J Qual Stud Health Well-being. 2024 Dec 17;20(1):2439467. doi: 10.1080/17482631.2024.2439467 (PMC11656756; doi:10.1080/17482631.2024.2439467)
Supplement: Biographical note.docx [file ZQHW_A_2439467_SM7655.docx]

**Biographical note**

Dr Tom Allport is a consultant paediatrician for Sirona Care & Health in Bristol’s Community Children’s Health Partnership, and honorary senior lecturer in the Centre for Academic Child Health in the University of Bristol. Working with children of forced migrant families in Bristol experiencing developmental difficulties has led him to study the experiential and interactional worlds of migrant families in British cities, and the ways in which ‘place’ can shape activity and opportunity. From this has emerged ideas and interventions termed ‘Find your village’, to support those who come to the ‘Global North’ from cultures where ‘it takes a village to raise a child.

Dr Hannah Briggs is a junior doctor developing interests in women’s, children’s and family health. As a medical student she studied Global Health for an intercalated BSc degree and would like to continue this interest in her developing clinical practice.

Professor Fatumo Osman is a senior lecturer and associate professor of nursing at Dalarna University in Sweden. Having researched migration's effects on parenting and family health for more than ten years, she has collaborated closely with migrant families, schools, and social services throughout her career. As a refugee who came to Sweden as a child, her work has primarily been inspired by epistemic injustice. By focusing on migration, mental health, parenting, and creating culturally tailored parenting supports and inclusion, she has become a leader and fearless advocate for migrants and refugees worldwide. Her current research is centered on women and adolescents' mental health and access to mental health services.
